# Sarcoma Spheroids and Organoids—Promising Tools in the Era of Personalized Medicine

**DOI:** 10.3390/ijms19020615

**Published:** 2018-02-21

**Authors:** Gianluca Colella, Flavio Fazioli, Michele Gallo, Annarosaria De Chiara, Gaetano Apice, Carlo Ruosi, Amelia Cimmino, Filomena de Nigris

**Affiliations:** 1 Division of Orthopedic Surgery, Department of Human Health, Federico II University of Naples, 80133 Naples, Italy; glc.colella@gmail.com (G.C.); Carlo.ruosi@gmail.com (C.R.); 2 Division of Musculoskeletal Oncology Surgery, National Cancer Institute, Pascale Foundation, 80131 Naples, Italy; F.fazioli@istitutotumori.na.it (F.F.); miga76@alice.it (M.G.); 3 Division of Pathology, National Cancer Institute, Pascale Foundation, 80131 Naples, Italy; anna.dechiara@istitutotumori.na.it; 4 Division of Medical Oncology, National Cancer Institute, Pascale Foundation, 80131 Naples, Italy; g.apice@istitutotumori.na.it; 5 Institute of Genetics and Biophysics “A. Buzzati Traverso”, National Research Council (CNR), 80131 Naples, Italy; amelia.cimmino@gmail.it; 6 Department of Biochemistry Biophysics and General Pathology, University of Campania “Luigi Vanvitelli”, 80138 Naples, Italy

**Keywords:** spheroids, tumor microenvironment, sarcomas, precision medicine, personalized medicine

## Abstract

Cancer treatment is rapidly evolving toward personalized medicine, which takes into account the individual molecular and genetic variability of tumors. Sophisticated new in vitro disease models, such as three-dimensional cell cultures, may provide a tool for genetic, epigenetic, biomedical, and pharmacological research, and help determine the most promising individual treatment. Sarcomas, malignant neoplasms originating from mesenchymal cells, may have a multitude of genomic aberrations that give rise to more than 70 different histopathological subtypes. Their low incidence and high level of histopathological heterogeneity have greatly limited progress in their treatment, and trials of clinical sarcoma are less frequent than trials of other carcinomas. The main advantage of 3D cultures from tumor cells or biopsy is that they provide patient-specific models of solid tumors, and they overcome some limitations of traditional 2D monolayer cultures by reflecting cell heterogeneity, native histologic architectures, and cell–extracellular matrix interactions. Recent advances promise that these models can help bridge the gap between preclinical and clinical research by providing a relevant in vitro model of human cancer useful for drug testing and studying metastatic and dormancy mechanisms. However, additional improvements of 3D models are expected in the future, specifically the inclusion of tumor vasculature and the immune system, to enhance their full ability to capture the biological features of native tumors in high-throughput screening. Here, we summarize recent advances and future perspectives of spheroid and organoid in vitro models of rare sarcomas that can be used to investigate individual molecular biology and predict clinical responses. We also highlight how spheroid and organoid culture models could facilitate the personalization of sarcoma treatment, provide specific clinical scenarios, and discuss the relative strengths and limitations of these models.

## 1. Introduction

Sarcomas are a highly heterogeneous group of solid tumors originating from mesenchymal stem cells (MSCs) [1]. MSCs are multipotent precursor cells of mesenchymal tissues, such as bone, cartilage, fat, and muscle. Based on the wide variety of sarcoma subtypes, the origin of sarcomas can be explained by alterations in MSC-committed cells. Their incidence varies from 3.3 cases per 100,000 in Eastern Europe to 4.7 per 100,000 in Northern Europe [2,3] and they account for 15% of all cancers in childhood and adolescence [4]. The five-year survival rate depends on the type, stage, and location, and the age of the patient. It is reported to be about 60% when diagnosed in early stages [2] but dramatically drops to 10% in advanced stages [5]. Given the heterogeneity and complexity of sarcomas, their clinical management has not advanced nearly as fast as that of many other carcinomas. Clearly, a better understanding of human sarcoma oncogenesis, metastasis, and drug resistance is warranted. The availability of new technologies, such as next-generation sequencing and digital western blot, has improved the selection of novel prognostic molecular markers. However, the low incidence of sarcoma subtypes and insufficient case numbers of individual subtypes make it difficult to validate such markers. Consequently, only a small number of molecular markers are currently available for clinical use. The effect of sarcoma drugs is also affected by intratumoral heterogeneity and the microenvironment, which are important determinants of tumor malignancy and metastasis [6,7,8]. Any model used to define sarcoma subtypes and treatment efficacy must, as far as possible, address these limitations. Cell culture models of sarcoma suffer from the fact that available cell lines are limited to the most common groups, such as osteosarcoma, leiomyosarcoma, and rhabdomyosarcoma, whereas none are available for such subtypes as alveolar soft-part sarcoma and giant-cell tumor of bone [9]. Furthermore, the success rate of sarcoma cell isolation and long-term 2D culture is very limited, mainly because they do not attach well directly on plates and have high genomic instability, particularly aggressive phenotypes. Studies that used 2D cultured tumor cell lines often yielded conflicting results, indicating that culture conditions and the number of cell passages are important. Bruland et al. were the first to develop an alternative to the classical monolayer culture procedure, based on nonadherent cell cultivation. Using this method, they generated 11 sarcoma cell lines from a patient with a 50% success rate [10]. More recently, Salawu further improved this method, increasing the success rate and stability of long-term cell growth, thus making it suitable for studies of the progression of osteosarcoma [9]. Although 2D in vitro models are inexpensive and relatively easy to generate and maintain, they do not accurately reflect the solid tumor characteristics and the complex cross-talk between tumor cells and their microenvironment (Table 1). Therefore, researchers are currently developing novel patient-derived 3D tumor cultures to reproduce the molecular complexity of sarcoma carcinogenic mechanisms and the environment, and to increase sensitivity to pharmacologic treatments. Currently, 3D models are widely used to model different cancers, including breast [11], cervical [11], colon [12], lung [13], pancreatic [14,15], and prostate [16]. Here, we review three-dimensional models of sarcoma that have been recently proposed to study the impact of the tumor microenvironment on its pathogenesis, growth kinetics, and gene expression, and for drug testing. We discuss current applications and future directions, focusing on sarcomas. However, this also may be of interest for other forms of cancer that metastasize to bone and may be applicable to rare cancers.

## 2. Three-Dimensional Models of Tumors

### 2.1. Tumor Spheroids

Tumor spheroids are spherical aggregates of tumor cells that are self-organizing and self-renewing. They may originate from single-cell suspensions of tumor cell lines, patient-derived tumor cells, or tumor stem cells cultured in nonadherent substrates [17]. In addition to tumor cells, endothelial cells, and possibly also mesenchymal cells and monocytes, may be included in the co-culture to better sustain and model the tumor microenvironment [18] (see paragraph below). The resulting tumor spheroids have three distinct zones: (1) a central core of necrotic cells, (2) an inner layer of nonproliferating quiescent cells, and (3) an outer nutrient-rich layer of proliferating cells interacting with the surrounding extracellular matrix (ECM) [19]. Although one cannot exclude that established culture conditions may be suitable for particular subpopulations of tumor cells, spheroids more closely reflect the phenotypic behavior of original tumors than conventional cell cultures [20]. Generation of spheroids is particularly important for sarcomas, because growth rates, cell morphology, cell–cell junctions, and kinase activation of spheroids closely mimic those of primary tumors [21]. Their potential use ranges from tumor drug tests [22,23,24,25] and cell proliferation studies [21,26] to investigations of the role of the microenvironment [27,28]. Furthermore, stem cell spheroids have proven useful for modeling micrometastatic disease and have contributed to our understanding of tumor anoikis, a form of programmed cell death that occurs upon loss of ECM contact [29,30]. Great effort is being expended to develop spheroids in co-culture with endothelial cells, to better study osteosarcoma angiogenesis and metastatic progression [31,32]. Co-cultures composed of tumor spheroids and mesenchymal stem cells are also being used to test novel compounds against tumor dormancy [33,34]. The methods that yield tumor spheroids can be classified into two broad categories: anchorage-dependent and -independent (see Table 1). Anchorage-dependent models utilize engineered scaffolds designed to simulate the ECM and provide structural or physical support. Among the most widespread materials are hydrogels containing proteins and ECM components used to encapsulate cancer cells in microporous scaffolds that mimic the native ECM and enable cells to adhere, proliferate, spread, and migrate in 3D. Generally, hydrogel derivative materials limit the penetration of compounds and exhibit considerable batch-to-batch variability. Some of these limitations may be overcome by the use of covalently modified synthetic hydrogels, often based on polyethylene glycol. Polyethylene glycol dimethacrylate hydrogel microarrays produce homogeneous spheroids with a median size of 50 μm in two to three days. Recently, several groups have developed 3D tumor models in hydrogel scaffolds and used them to assess the potency of a small number of cancer drugs or for small pilot screens of 1500 compounds [35].

Anchorage-independent models are based on the use of mechanical equipment that induces turbulence in the cell culture medium and prevents cells from adhering to solid surfaces or altering the cell–ECM interaction [36]. Another approach is based on the use of nonadherent microculture plates or various semisolid gel-coated Petri dishes that prevent cell binding, or the use of hanging drops [37,38] or microfluid chips [39]. Although these techniques potentially generate thousands of consistent copies of suitable spheroids, not all tumor cell lines spontaneously form 3D spheroids under these conditions [40,41]. Furthermore, many of these methods (e.g., agarose Petri dishes, hanging drops, and rotary cell cultures) generate irregular 3D cellular aggregates with a wide range of morphologies and sizes, whereas regular size is obtained with microfluid chips (see Table 1). More importantly, the time to obtain colonies ranges from one to two weeks (based on tumor type and method) when using 10% fetal calf serum in media, as suggested for single cell lines, and media need to be changed at least two times per week [42]. Moreover, some of these anchorage-independent 3D tumor spheroid methods are rather labor-intensive (e.g., microfluid chips and rotary cultures). Ultra-low-attachment microplates (ULA-plates) are the most promising approach [41]. These plates are coated with a hydrophilic gelatin, are neutrally charged, and support the growth and formation of tight spheroids, compact aggregates, or loose aggregates for a variety of human tumor cell lines. Tumor spheroids formed in 96-well U-bottomed ULA-plates exhibit similar morphology and immunohistochemical staining as spheroids grown in agar, and could be used for tumor cell migration and invasion assays [41]. With this technique, hundreds of homogeneous spheroids can be produced within two to three days in regular tumor cell media. It is therefore currently being adopted for high-throughput drug screening.

### 2.2. Tumor Organoids

**Figure 1:**
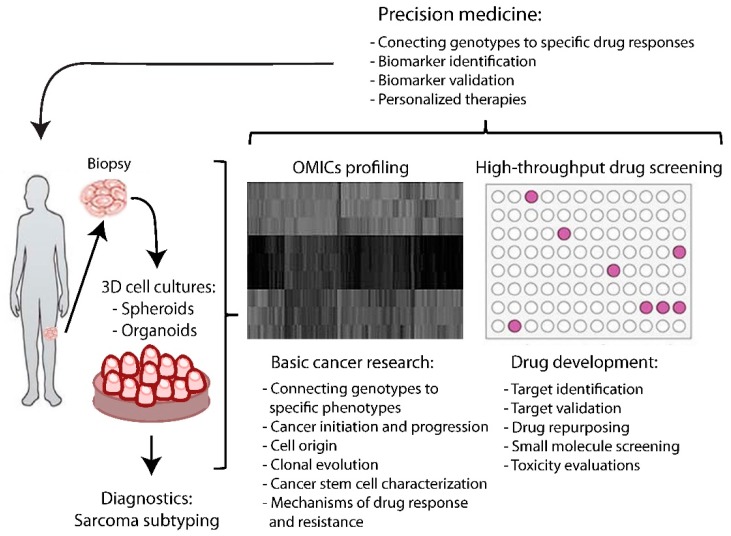
Workflow from biopsy to personalized medicine. From biopsy, it is possible obtain organoid and spheroid models that are sources of patient-specific DNA, RNA, and proteins (omics profiling). Patient omics may help in connecting genotype to phenotype, in order to select specific mutations, genes, and proteins and identify targets. Spheroids may be directly used for patient drug sensitivity screening and target validation. Integrating omics data and high-throughput drug screening can provide specific molecular and clinical scenarios for better personalized therapy.

Organoids are three-dimensional models of primary tumor tissue obtained from fresh biopsies. Organoids are generated by mechanical or enzymatic digestion of the original tumor section into small pieces. These are embedded in an extracellular matrix, such as Matrigel, or different types of collagen, where cell–cell interactions predominate over cell–substrate interactions and resemble avascular tumor nodules, or micrometastases, and mimic growth kinetics, gradients of nutrient distribution, oxygen concentration, and cell proliferation. Studies have demonstrated that ultra-low-attachment microplates generate organoids of larger size (300–400 μm), one per well, in a few days (three days if at least 5 × 10^3^ cells are plated), but they were never tested in sarcoma [43]. Organoids retain the structure, morphology, stromal composition, genetic mutations, and heterogeneity of the original tumor [44]. Furthermore, when organoids are transplanted into mice, they form carcinomas that histologically resemble the tumor of origin [44,45]. The opportunity to reproduce in vitro and within a few days both the complex structure of the microenvironment and the chromosome aneuploidies characteristic of individual sarcomas is of great interest, mainly because the environment changes the tumor’s cellular responses to drugs. Numerous assays have been employed to study aspects of 3D tumor organoids, including genetics, biochemistry, and imaging. These techniques have also been used to characterize the effect of anticancer therapeutics in 3D organoids [44,46]. Based on these studies, organoids represent a suitable platform for testing potential drugs on patient-specific tumors. The hypothetical workflow for a clinical organoid screen would be: (1) tumor biopsy, (2) organoid generation and growth, (3) genetic and drug panel screening, (4) response assessment, and (5) selection of the optimal drug for treatment of the patient (Figure 1). The entire process can be completed in a few weeks. This approach overcomes many of the limitations of traditional cancer models and patient-specific xenograft tumors in immunocompromised mice, in particular the high cost, low take-rate, and multi-month experiments required to obtain drug response data [47,48].

## 3. Implementation of 3D Microenvironments (Hybrid Models for Investigating Angiogenesis, Influence of Immune Cells, and Tumor Dormancy)

Many papers have discussed the differences between two- and three-dimensional cultures and emphasized that the latter model the tumor microenvironment. Several groups have proposed three-dimensional models in which endothelial and fibroblastic cells are co-cultured in the presence of substrates derived from human tumors, such as basement membrane and/or gels rich in collagen or laminin [49,50,51]. One of the most suitable is based on the use of ultra-low-attachment (ULA) plates in combination with the new technologies of automated imaging and analysis. The method consists of plating monolayers of endothelial cells or fibroblasts from different sources and letting tumor cells self-assemble. The ratio between endothelial and tumor cells is generally 1:1. In five days, endothelial cells begin to differentiate, as indicated by the presence of CD34 antigen, and sprout in the inner layer of spheroids [39]. Co-cultures of breast cancer spheroids and human umbilical vein endothelial cells (HUVEC) have already been used to test anticancer drugs targeting epidermal growth factor receptor [52]. However, each tumor type generates different interactions with its neighboring cells and endothelial cells, suggesting the need for a platform for personalized medicine that would account for these differences [53].

Chaddad et al. combined, for the first time, a monolayer of HUVEC with osteosarcoma 3D MG-63 cells using hanging drop techniques. Within five days, co-cultured 3D spheroids expressed extracellular matrix proteins, such as osteocalcin and osteopontin, and led to the establishment of an organized architecture similar to that of tumors encountered in vivo. Furthermore, MG-63 cells cultured in 3D spheroids expressed more bone proteins and vascular endothelial growth factor (VEGF) than in monolayer culture. The architecture of co-cultured spheroids showed cellular activity at the periphery and quiescent cells embedded in matrix at the center of the spheroid. Moreover, the hypoxic core favored the secretion of VEGF, which attracts endothelial cells to the spheroid tumor and promotes formation of vessel-like structures and organization of a vascular network [54]. Spheroids also indicated that the skeletal vascular microenvironment is involved in controlling the fate of tumor cells, active and dormant, and can influence multiple steps within the metastatic cascade [55]. Hybrid models composed of tumor cells, fibroblasts, and immune cells are a novel hot topic. They were primarily developed to investigate reciprocal interactions of regulatory T cell lymphocytes and natural killer cells with luminal and basal phenotype breast cancers [56]. Furthermore, they seem to provide novel efficacy tools for in vitro evaluation of cancer immunotherapy agents, and for investigation of immune cell infiltration and drug targeting [57]. However, they have not yet been tested for sarcomas. Another application of co-cultures and three-dimensional models expected to have a great impact in the near future is the use of mesenchymal stem cell and tumor spheroids to study tumor dormancy [34]. Bone marrow disseminated tumor cells are dormant cancer cells that are harbored in bone marrow niches for years after cancer remission, before potentially returning to a proliferative state and causing cancer recurrence. Currently, there are very few models that recapitulate the dormant phenotype. Buschhaus and co-workers recently used a three-dimensional spheroid-based model co-cultured with mesenchymal stem cells and integrated dual-color bioluminescence imaging to quantify differential cell viability in response to various compounds. They successfully screened for compounds that selectively eliminated cancer cells versus supportive stromal cells, and validated the results by comparing them to efficacy in vivo [34].

## 4. Bone Tumor Niche

Bone constitutes the microenvironment of several groups of sarcomas and modulates primary tumor growth and metastasis [58]. To recapitulate their features in vitro, several bioengineering methods have been developed to improve 3D models [58]. In bone, mechanical forces generated either from external sources or by cell contractions play important roles in cancer invasion of the bone [59]. Three-dimensional models reproduce the architectural, mechanical, and biochemical elements of the bone microenvironment [60]. These models combine tumor cells from patients and bone scaffolds from different sources [61]. In some models, the scaffold consists of decellularized bovine bone precoated with human mesenchymal cells, a peculiar system that allows prolonged culture (four weeks) [62]. Cell proliferation within scaffolds more closely approximates the real Ewing sarcoma cell growth rate, which is significantly less than that of monolayer cultures [63]. Scaffolds also reproduce the physiological exchange of nutrients, oxygen, and metabolic waste byproducts, and ideally are compatible with standard experimental techniques (e.g., microscopy, immunohistochemistry, cell proliferation assays) [64]. A three-dimensional bone model of Ewing sarcoma was shown to maintain the immunohistochemical biomarkers normally expressed (CD99+, IGF-1R+, keratin−, and SMA−) [65]. Furthermore, proteomic expression profiles along the IGF-1R/PI3K/mTOR signaling pathway suggest that 3D scaffolds can reliably mimic critical signaling cascades in human Ewing sarcoma tumors [66]. Similar observations were made in breast cancer metastasis cells grown in 3D cultures [67]. This bioengineered human tumor model can potentially improve upon the current preclinical drug-screening paradigm by providing valuable information about new therapeutic targets and anticancer drug efficacy in primary bone tumor and metastasis. However, one of the most important questions yet to be resolved is whether the 3D bone model shares enough hypoxic fidelity to be useful as a preclinical high-throughput drug testing platform. Hypoxia per se stimulates blood cell proliferation and blood vessel formation, and modulates the expression of extracellular matrix components. To date, only a few reports have considered bone hypoxic conditions in preclinical studies and three-dimensional in vitro models [68].

## 5. Applications for Sarcoma Biology Research

### 5.1. Functional Genomic Analysis

The sarcoma genome is complex, with a large number of chromosomal variations and a relatively small number of recurring exonic mutations [69]. Some mutations and genomic rearrangements contribute diagnostic information and may help to identify targeted treatment [69]. Assessing drug effects in 2D cultures is difficult, mainly because the small number of available tumor cell lines cannot represent the genetic diversity of highly heterogeneous sarcomas. Such cancer cell lines are generally limited to the most common subtypes, and most of the cell lines available were isolated from very aggressive tumors that do not represent low-grade tumors. Specific cell lines or cells isolated from a small number of patients therefore cannot truly represent the pathology, and data from a larger number of tumors and more diverse tumors are necessary to establish their carcinogenic mechanisms and drug responses. In recent years, patient-derived xenograft (PDX) models have proven useful to study the histology of some sarcomas [70]. For these PDX models, a tumor mass from a patient is directly implanted into the subcutaneous tissue of a severe combined immunodeficiency mouse, which is followed by tumor growth. Upon reaching a sufficient mass, tumors are explanted, divided into smaller samples, and reimplanted for subsequent passage in mice. Therefore, a small piece of tumor can be propagated to achieve a tumor mass manyfold greater than that of the original tumor. Comprehensive genomewide gene expression analyses have shown that tumors from PDX models at an early passage have expression profiles that are very close to those of the original tumors [70]. Some investigators have integrated PDX with in vitro genome profile to identifying factors that underlie heterogeneous patient responses, and have thus identified associations between a genotype and drug response, as well as mechanisms of resistance in breast cancer [47]. This approach could therefore be useful for personalized patient treatment, especially in tumors with low numbers of mutations, such as gastrointestinal stromal tumor sarcomas. The same approach, however, is less practicable for some tumors with higher chromosome instability and a prominent involvement of immune cells, such as giant-cell tumors of bone.

One opportunity for such sarcomas could be the use of multicellular spheroids and organoids from patients to assess both genetic profiling and drug responses. This combination therefore provides a promising approach to assess the relevance of specific mutations and to test patient-specific drug responses at the bedside. In fact, sequences of spheroids from sarcomas showed that they retain the genetic signature of the patient’s tumor and are a readily available source of high-quality DNA and RNA for next-generation sequencing [71]. Furthermore, gene-specific silencing may be introduced in spheroids by different techniques, such as small interfering RNA or CRISPR-Cas9, in order to assess their functional role in the pathogenesis of sarcomas [72,73]. The latter approach can be particularly useful to study genetic variants, epigenetic alterations, or even changes in chromatin structure, and to develop specific treatments. For example, Hanes and colleagues selected targetable substrates based on tumor genomic profiling. Using patient-derived cell lines, they first profiled the genomes and selected a target. They then tested cell sensitivity to target-specific drugs in vitro, and thus demonstrated the benefit of combining tumor genomic profiles and in vitro drug testing [74]. These results also support the idea that better classification of patients based on a personalized model could help in choosing the best treatment for sarcomas [75]. Proteomics represents another molecular approach to selecting biomarkers of sarcoma progression and therapy. Several research groups have focused on differential expression of proteins in tumor tissues, using various approaches [74,76], and stratified patients based on histology and grade [77]. Toward this goal, a multi-institutional consortium has been established that will provide in-depth analysis of existing genomic databases, proteomic data analysis, and statistical support, and prioritize and track the discovered targets.

However, these integrated approaches do not select therapies, nor do they indicate the best treatment for a single patient. Spheroid models offer the opportunity to profile both genomic and proteomic profiles individually and to determine the best drug from the same biopsy. Therefore, they seem to be a more promising way toward more effective treatment of rare sarcomas.

### 5.2. Preclinical Drug Screening

As previously emphasized, the drug sensitivity of tumor cells is strongly affected by microenvironmental factors [78,79]. The spheroid approach is of special interest for the treatment of bone sarcomas, which are particularly drug resistant due to the presence of the bone barrier and tumor heterogeneity [80]. As proof of concept, a multicellular spheroid model of osteosarcoma was used to evaluate the anticancer efficacy of VOchrys compared to cis-platinum [81]. In this study, a vanadium (IV) complex with the flavonoid chrysin displayed better effect than cis-platinum in osteosarcoma spheroids, because VOchrys altered the shape of spheroids and decreased their viability. The pharmacological effects were also confirmed in a xenograft model, which showed a significant reduction of tumor size. These results demonstrate that spheroid models can be predictive of drug activity and that it is possible to measure drug efficacy in multiple cells that compose osteosarcomas [81].

Another particular aggressive and drug-resistant sarcoma type is the chondrosarcoma. The chemoresistance of cartilage tumors results from phenotypic microenvironmental features of the tumor tissue, mainly the chondrogenic extracellular matrix (ECM) and hypoxia. In an interesting study by Voissiere et al., both 2D and 3D chondrosarcoma models were tested with doxorubicin alone or in combination with TH-302, a pro-drug activated in hypoxia [25]. Spheroids developed much earlier resistance to doxorubicin than two-dimensional cell cultures, due to a limited penetration of the drug into the deeper layers of spheroids as a result of cell adhesion between tumor and stroma cells in the 3D systems [82,83]. In contrast, spheroids were sensitive to TH-302. Interestingly, larger spheroids were more sensitive to TH-302 (multicellular resistance index (MCRI) = 7.7) than smaller hypoxic spheroids (MCRI = 9.1) [25]. The authors speculated that the drug resistance could also be due to the presence of layers of cells in different stages of the cell cycle [84]. It therefore seems that a better understanding of the molecular pathways governing cell adhesion and ECM in sarcomas would be of interest and may lead to the identification of new targets in the stroma, including stromal cells, ECM entities, matrix-degrading proteases and inhibitors, and regulatory substances [85]

Most studies on spheroids as models for in vitro tumor drug tests indicate several advantages (also see Table 1). The first is the possibility to generate, from a single biopsy, multiple spheroids identical in structure, morphology, and microenvironment. The second advantage is that drug response can be measured in microenvironments or on cell subpopulations isolated from different spheroid regions [40,86,87,88,89,90]. Spheroids cultured on confluent endothelial cells were used to test drugs that affect tumor cell evasion and sprouting phenomena in vitro [91]. Incubation of spheroid cultures with immune cell suspensions is another interesting approach to mimic local immune responses, and to test penetration, local distribution, and the effect on tumor cells of antibody-based treatments [92,93]. The third advantage is that the spherical symmetry simplifies the mathematical analysis to predict radiation response [94], drug penetration, and binding/activity [30,94,95]. Another area of interest is the use of spheroids derived from normal untransformed cells, such as hepatocytes/liver [96,97] or chondrocytes/cartilage [98], to study organ or tissue development, or to evaluate drug selectivity and specificity, by comparing them to tumor spheroids [86]. Following drug uptake in individuals, spheroids could also be used to evaluate the effective diffusion coefficients. Analogous to monolayer cellular assays, easy multiwell systems are available to screen drug effects, such as cytotoxicity, proliferation, binding, apoptosis, and adenosine triphosphate levels in spheroids [99].

Given the similarities between results in 3D models and xenographs, pharmaceutical companies have developed platforms and chips to screen several different therapeutics. Large-scale 3D tumor spheroids represent a preclinical testing pipeline to determine a drug candidate’s activity, toxicity, and pharmacokinetic profile in high-throughput drug screens [100]. Additionally, they may also help to reduce side effects. Furthermore, maintaining multiple organoids from the same patients in culture allows later investigation of treatment-induced selection processes and potential resistance mechanisms [101] to be carried out. A preliminary analysis of the SpheroNEO breast cancer study cohort (172 patient spheroids) showed a predictive response to trastuzumab-based therapy, but was also selective in discerning ineffective from effective treatment options [102].

Some limitations of laboratory methodology have to be acknowledged. The main difficulty is obtaining the minimal number of cells required for a reliable assay outcome from surgical specimens that vary from patient to patient and depend on the anatomical location. The minimum number of tumor cells used in all studies cited here was 5000. Furthermore, the methods have to be customized based on tumor histology. Ultra-low-attachment plates seem to be the best to obtain a homogeneous size of organoids in four to five days. From a clinical standpoint, cancer spheroids have to be stable for two to three weeks in order to be able to perform all screening. This may not always be possible, except for engineered bone scaffold models that can survive for one month. In this regard, it is noteworthy that several groups are working to establish organoid banks [103,104,105].

Three-dimensional models to assess patients’ individual responses to radiotherapy may also become a future tool for personalized treatment [99,100,101]. Chemoradiation currently serves as first-line therapy for advanced and chemoresistant chondrosarcoma, while new chemotherapy regimens are emerging. However, response rates for any treatment are difficult to predict and have broad variation, which could be improved by using 3D models.

## 6. Clinical Opportunities

Several studies have indicated that short-term 3D cell cultures have superior predictive value in the preclinical arena over in vitro models and are comparable to xenographs. Therefore, several cancer centers around the world have started to determine patient-specific 3D cell culture data [106]. Some have explored the molecular characteristics of individual patients’ tumor spheroids or organoids, using them as a platform for drug screening in an automated setup to identify new biomarkers and potential drug targets [107]. Because the screening, integration of genotype-specific drug discovery, and in silico validation take a few weeks, these approaches have the potential to select the most appropriate individual treatment concurrent with the initial treatment and may then influence further therapy. However, it should be emphasized that personalized medicine could not only offer therapeutic value to patients and their oncologists, but at the same time help to gain a better understanding of their specific tumors. Although a perfect preclinical cancer model may be unattainable within the limitations imposed by the amount of fresh tissue that can be safely obtained, spheroid models greatly expand the spectrum of cancer subtypes that can be modeled and offer new possibilities for drug discovery and personalized medicine for rare cancers, such as sarcomas.

## Figures and Tables

**Table 1:** Comparison of various tumor models.

Models	General Properties/Advantages	Cost and Reproducibility	Disadvantages	References
**2D cell cultures** Monolayers cultured on polystyrene or other adherent flat surfaces	- Cellular heterogeneity: Different cell types can be present (co-cultures) - Extracellular matrix (ECM): not physiological - Gene expression: Most genes and mutations of tumors are expressed in early passage	- Low cost; - High reproducibility	- Do not mimic in vivo 3D organization of tumors - Faint or undetectable expression of ECM proteins (collagens, fibronectin) - Poor ECM-cell interactions - Gene expression levels differ from those of in vivo tumors - Drug testing: lack of drug penetration barriers; drug resistance	[108,109]
**3D scaffold-based cell cultures** Cells seeded in structures of different materials: hydrogels, 3D life biomimetic, 3D Insert™ scaffolds (synthetic bone derivatives), Alvetex^®^	- Cellular heterogeneity: Different cell types can be grown in the hydrogel; cellular 3D organization occurs spontaneously; necrotic zones may be formed - Gene expression: Gene expression and cell composition similar to in vivo tumors - Drug screening: Suitable to investigate drug penetration and interactions of cells with bone derivatives; spheroids obtainable in 2–7 days - Useful to study hypoxia	- Some expensive materials and special equipment required; - Extensive handling necessary, time-consuming; - Reproducibility is highly dependent on the technique used to produce scaffold	- The size of spheroids is not homogeneous and is generally around 50 µm - The ECM formed is artificial - Some cell types grow better than others - Not suitable for studies of matrix invasion and cell–cell interactions - Not useful for high-throughput drug screening - Long-term stability and survival are poorly known	[109,110,111]
**3D nonscaffold-based cell cultures** Spheroids from single or multiple cell types; Low-adhesion plates; Micropatterned surfaces (Scivax NanoCulture^®^ multiwell plates); Hanging drop plates (Perfecta3D^®^, Gravity-PLUS^™^ 3D)	- Cellular heterogeneity:Different cell types can be used for spheroid production; Presence of a necrotic core and peripheral layer of cells with a high proliferation rate; Suitable to study cell–cell contact and cell invasion - Gene expression: Gene expression and cell phenotype are very similar to in vivo tumors; Useful to study genotype and omics - Drug screening: Cell–cell interactions and high ECM density are responsible for impaired drug response in vivo; Useful to predict patient response to drugs; May also be useful to study immuno- and chemokine drugs	- The majority of techniques currently used are expensive and allow production of a large number of spheroids - Spheroids with a median size of 300–500 μm can be obtained in 2–3 days This is sufficient for organization of cells in three layers: necrotic layer; median layer, low growth rate and low pH; high-growth-rate layer - Correlation of drug efficacy results with clinical results yet to be established - Methods available to store as bank	- Deposition of ECM may be similar to that in tumors - Gene expression and phenotype similar to in vivo tumors - Ratio of different cells in multiple-cell spheroids needs to be established - May not reflect differences in oxygenation resulting from blood perfusion - Limited modeling of immune mechanisms - Optimal growing conditions need to be established for different tumors - May not reflect differences in oxygenation resulting from blood perfusion	[108,109]
**Animal models** Patient-derivative xenograph (PDX)	- Closest to human - Allow maintenance of intratumoral heterogeneity - Useful to monitor effects of neovascularization - Needed to detect broader drug effects - Useful to study genomic and omic’s profiling of human biopsies	- High cost and need for animal facilities - Ethical concerns and prohibitive costs rule out primate models	- Do not reflect complex immune and metabolic environment - Not necessarily representative of human cancer and therefore of limited predictive value - Significant anatomical, immune, and metabolic differences in men - Low take-rate and multimonth experiments for drug response data	[47,70]
